# Effect of Employees’ Perceived Green HRM on Their Workplace Green Behaviors in Oil and Mining Industries: Based on Cognitive-Affective System Theory

**DOI:** 10.3390/ijerph18084056

**Published:** 2021-04-12

**Authors:** Silu Chen, Wanxing Jiang, Xin Li, Han Gao

**Affiliations:** 1 School of Economics and Business Administration, Central China Normal University, Wuhan 430079, China; chensilu@mail.ccnu.edu.cn; 2 School of Business Administration, Shanghai Lixin University of Accounting and Finance, Shanghai 201620, China; 3 School of Urban Design, Wuhan University, Wuhan 430072, China; li-xin@whu.edu.cn; 4 School of Architecture and Civil Engineering, Xiamen University, Xiamen 361005, China; 5 College of Fashion and Design, Donghua University, Shanghai 200051, China; gaohan708@dhu.edu.cn

**Keywords:** perceived green HRM, green psychological climate, harmonious environmental passion, voluntary workplace green behavior, green creativity

## Abstract

Drawing on cognitive-affective system theory, this study proposes that employees’ perceived green human resource management (HRM) influences their’ workplace green behaviors through two psychological processes: the cognitive and the affective route. By analysing 358 questionnaires collected from Chinese firms in the oil and mining industry, we obtain evidence in support of our predictions, finding that employees’ perceived green HRM positively impacts their voluntary workplace green behaviors and green creativity. Additionally, green psychological climate and harmonious environmental passion are found to partially mediate the relationship between employees’ perceived green HRM and voluntary workplace green behavior while harmonious environmental passion is found to fully mediate the relationship between employees’ perceived green HRM and green creativity. These findings shed light on the importance of green HRM in shaping employees’ proactive workplace green behaviors and uncover how green HRM transforms employees’ cognitive, affective, and motivational (CAM) factors into green actions.

## 1. Introduction

At present, economic development has inevitably brought about issues related to environmental pollution in China, including climate change, sewage discharge, and biodiversity loss. Such environmental issues are directly or indirectly caused by people’s daily behaviors [1]. As organizations draw essential inputs from the natural environment, the sustainability of business depends greatly on their treatment of the natural environment [2]. Research [3,4] has found that organizations can generally increase their levels of branding recognition and sales by having a set of green policies in place. Employees, as the agents who actually implement such green practices, play an important role in helping an organization achieve its corporate green goals [5]. Given that human resource management (HRM) is mainly presented in relation to the environmental bottom line [6] and HRM practices play a critical role in dictating whether employees feel comfortable exhibiting their proactive tendencies [7], organizations have increasingly adopted green HRM practices to promote employees’ green behaviors, such as organizational citizenship behaviors (OCB) towards the environment [8], task-related and voluntary employee green behaviors [9], eco-friendly behavior [10], and proenvironmental behavior [11]. Green HRM can be defined as “HRM aspects of green management”, to promote employee green behavior in the workplace [12] (p. 1). Despite a recent surge in green HRM research, an understanding of how HRM is perceived by employees and affects their attitudes and behaviors remains limited. For example, little is known about whether and how green HRM shapes prospective employee outcomes from both cognitive-affective frameworks. Even though the current literature has documented the antecedents of employees’ workplace green behaviors, including individuals’ values and behavioral intentions [13], corporate environmental responsibility [14], corporate social responsibility [15], daily affect [16], and transformational leadership [17], little work has focused on the effect of formal organizational context (i.e., HRM). In fact, compared to individuals’ characteristics or leadership styles, which are usually stable and hardly change over a short period of time, green HRM as an instrumental strategy helps organizations to achieve their sustainability-oriented goals through creating a green environmental culture and green employees who are concerned about environmental issues [6,11,18]. Moreover, some HR literature [19] suggests that different HRM practices may influence employees’ behavior through different psychological processes, however, it still remains unclear how green HRM influences employees’ green behavior in the workplace. Therefore, it would be of great significance to focus more on the effect of the organizational situation factor, i.e., the green HRM on employees’ proactive green behaviors as well as on the mediating process about how such HRM practices exert important influence on employees’ workplace green behaviors.

This paper focuses on employees’ proactive workplace green behaviors (i.e., voluntary workplace green behavior and green creativity). Voluntary workplace green behavior refers to “discretionary employee actions that contribute to the environmental sustainability of the employer organization but are not under the control of any formal environmental management policies or system” [20] (p. 3). It can also be seen as an extra-role behavior in which employees use their initiative to engage in behavior that encourages sustainability in the work context going beyond their formal job-tasks [21]. According to Kim et al. [20], this behavior benefits organizations directly by conserving resources and energy for cost reduction and indirectly by preserving the natural environment for organizational sustainability. Besides, green creativity refers to ‘‘the development of new ideas about green products, green services, green processes, or green practices that are judged to be original, novel, and useful’’ [22] (p. 109). Nurturing green creativity among employees to produce innovative end results is the primary concern of managers [22,23]. To achieve sustainable development of environment, organizations heavily depend on the behaviors of their employees [24]. Thus, we purpose that voluntary workplace green behavior and green creativity are two importance outcomes of green HRM, which is beneficial to an organization’s operations.

This study intends to make three main theoretical contributions. First, it adds to the literature of green HRM by exploring the employee outcomes of green HRM in the workplace, thus help better understand the concept of green HRM as well as its consequences. As an emerging research topic of HRM, green HRM has not been sufficiently explored in terms of its effect on employees’ workplace outcomes. Even though past research mostly reached the conceptualization stage [12,25], empirical testing and theorizing of the effect of green HRM on employees’ workplace green behaviors is still in its infancy. Our study thus contributes this stream of research by empirically testing the effect of employees’ perceived green HRM on their green behaviors, especially such effect in the context of the oil and mining industry in which the companies are very environmentally sensitive in nature and employees’ proactive green behaviors are encouraged and valued.

Second, past research suggests that HRM practices might not directly exert influence on employee outcomes in the workplace, but indirectly does so through certain path or some psychological processes [19]. However, such a mediation path has been merely studied. Our research therefore contributes to these streams of research and enriches the literature on the psychological processes of individuals’ green behaviors such as employees’ green psychological climate and harmonious green passion. Responding to the call of Renwick et al. [12] for better understanding of the underlying mechanisms between organizations’ practices and employees’ green behavior, our research fills such major gap in the existing literature, thus making an indispensable contribution theoretically.

Last but not least, existing research explained the influence mechanism of green HRM on employees from various theoretical perspectives, such as the theory of planned behavior (TPB) [24], supplies-values fit theory [26,27], social identity and stakeholder theory [11,28], social exchange theory [29], Ability-Motivation-Opportunity (AMO) and contingency theory [30,31], but few studies have examined the mechanism from the cognitive-affective system theory. In fact, cognitive-affective system theory encompasses important psychological determinants of behavior within one dynamic system and provides a more detailed conceptual model for people’s attitude and behaviors, thus can be a very helpful theoretical perspective in explaining how green HRM transforms employees’ cognitive, affective, and motivational (CAM) factors into green actions. However, this perspective has been absent from the current literature in the field of green HRM and environmental protection. Our current study, therefore, fills such a research gap and makes an important contribution theoretically.

The remainder of this paper is organized as follows. First, we develop our hypothesis based on cognitive-affective system theory. We then conduct an empirical study from the oil and mining industry and discuss the results, while detailing the associated methods. From there, we present the theoretical contributions and practical implications of this research. Finally, we summarize the current study by outlining its limitations and potential future research directions.

## 2. Theory and Hypotheses

### 2.1. Cognitive-Affective System Theory

Cognitive-affective system theory was first used to describe the dynamics between individuals’ personalities and related behaviors [32]. It states that individuals’ behaviors are influenced by their own characteristics. Cognitive-affective units are not isolated, but rather are interconnected and organized, guided by a stable network of cognitions and affects the characteristic for that individual [32]. An organization can be regarded as a unit, and individuals’ personalities and behaviors can be activated and processed dynamically through the cognitive, affective and motivational mediating processes that occur within such a unit [33]. Besides, cognitive-affective system theory assumes that individual differences in social behaviors tend to show some variability across different situations. In other words, the extent to which individuals exhibit different personalities and behaviors depends on the different units or organizations they are in, i.e., there exists a dynamic interactive effect between the environment and the individuals’ behaviors [34].

According to this theory, the personality system contains mental representations whose activation leads to the behavioral consistencies that characterize the person. These representations consist of diverse cognitive-affective units, which include the person’s construal and representations of the self, people, and situations, enduring goals, expectations, beliefs, and feeling states, as well as memories of people and past events [35]. Drawing on cognitive-affective system theory, in this study, we argue that it is possible that employees may exhibit different levels of green behaviors in the workplace based on the organizational situation factors as well as their different cognitive-affective psychological processes or psychological features they experience. In other words, the current study assumes that individuals’ levels of green behaviors can be influenced by the interactive effect among the cognitive, affective and motivational factors.

### 2.2. Green HRM and Employee Workplace Green Behavior

This paper measures green HRM from employees’ perceptions because differences in personal experiences and idiosyncratic preferences may lead employees to react differently to practices [36,37]. Existing studies have identified that green HRM can encourage employees to participate in practices beneficial to an enterprise’s sustainable development (e.g., electronic filing, virtual conferencing, online training or energy-saving office management) [38,39], as well as improve employees’ cognition and levels of commitment towards their organizations’ environmental agendas and then better address their organizations’ green goals and objectives [10]. Specifically, in the recruitment and selection process, green HRM focuses more on employees’ green values and tends to attract those who value sustainable development [40]. For instance, green and sustainable development agendas can be incorporated into job descriptions and organization descriptions. Interview questions related to green issues and an organization’s sustainable development can also be used to help select employees who exhibit potential and knowledge in terms of green issues [39]. With green HRM training, it helps increase employees’ environmental protection awareness [41], improve their skills and abilities in terms of environmental protection [42], enhance their sense of responsibility and motivation in regard to environment management and encourage their engagement and participation in green behaviors. As for performance appraisal, green HRM considers employees’ green performance in the appraisal, promotion and rewards process. For instance, bonuses, promotions and extra gifts can be given to employees who engage in green behaviors so that employees are more likely to be motivated to contribute to and engage in green activities [12,43]. For the employee involvement, green HRM focuses more on empowering employees in terms of green issues and should encourage and value employees’ suggestions regarding pro-environmental initiatives [44]. To sum up, green HRM incorporates “green” elements into these sets of practices in terms of recruitment, training, performance appraisals, and rewards, which can influence employees’ attitudes to elicit green behavior in the workplace. When employees perceive that their organization is adopting an openly communicated and formalized series of policies and practices that can overtly demonstrate their organization’s green commitment and goals [10], they are more likely to not only act in accordance with their organization’s green policies but to also engage in more voluntary workplace green behaviors and even green creativity. Hence, we hypothesize the following:

**Hypothesis** **1a.** *Employees’ perceived green HRM is positively related to employees’ voluntary workplace green behavior.*

**Hypothesis** **1b.** *Employees’ perceived green HRM is positively related to employees’ green creativity.*

### 2.3. The Mediating Role of Green Psychological Climate

In this study, we draw from cognitive-affective system theory and propose that employees’ perceptions of green HRM may transmit its influence on employees’ proactive green behaviors through two different paths (i.e., cognitive and affective routes). Cognitive attributes such as expectancies influence how individuals perceive cues and construct meaning of green HRM [45]. In particular, we argue that green psychological climates can serve as key psychological and social factors through which employees’ perceived green HRM influences their green behaviors in the workplace. Green psychological climate is developed from psychological climate and green climate. Psychological climate refers to how organizational environments are perceived and interpreted by their employees [46,47]. Green climate has been described in the literature as the climate that applies to corporations that achieve sustainable objectives by implementing a range of pro-environmental policies [18,48,49]. Green psychological climate, therefore, is the perception an individual has of the organization’s pro-environmental policies, processes, and practices that reflect the organization’s green values [26]. Research has shown that green psychological climate is positively related to employees’ green behavior [13,26,49].

Employees’ social interaction with their organizations will contribute to the psychological climate, and they can determine the value of organizational practices, procedures and policies that they observe or encounter [50], including green HRM. When an organization has a strong HRM system in place, employees may interpret and digest such a management style and in turn determine how they perceive such green values and their organization [36,51]. It is through such a cognitive process that employees gradually develop views regarding organizations’ green psychological climates. When an organization adopts a series of HRM practices, employees view the organization as concerned not only with economic gains but also with employees’ green-related activities and decisions [12]. In other words, organizations adopted green HRM and will incorporate these “green” elements into management areas by clarifying green responsibilities through job design and appraisals, rewarding green behaviors or promoting employee involvement in green behaviors. All of these strategies can help employees bring “green” to their attention and perceive a strong green psychological climate.

We argue that the green psychological climate perceived by employees could increase their willingness to make extra efforts beyond their duties. Prior research [52] suggests that an organization’s climate can predict its employees’ behavior and performance to some extent. For instance, employees may increase their overall effort in response to perceived concern for their climate safety [53]. Parker et al.’s [54] literature review also demonstrates that psychological climates are related to employees’ outcomes, including job burnout, job satisfaction and job performance. Specifically, Rupp et al. [55] found that employees’ perceptions of activities involving corporate social responsibility can trigger their attitudinal, emotional and behavioral responses. Similarly, Norton et al. [49] found that the association between employees’ perceptions of the presence of environmental policies and green behaviors, including proactive and task-related green behaviors, and green work climate perceptions constitute psychological mechanisms that link such policies with behaviors. Based on the above arguments, we hypothesize the following:

**Hypothesis** **2a.** *Green psychological climate mediates the relationship between employees’ perceived green HRM and employees’ voluntary workplace green behavior.*

**Hypothesis** **2b.** *Green psychological climate mediates the relationship between employees’ perceived green HRM and employees’ green creativity.*

### 2.4. The Mediating Role of Harmonious Environmental Passion

In addition to the cognitive mediating process of green psychological climates, employees’ perceived green HRM may influence employees’ green behaviors via an affective route. Affect refers to a broad range of feelings that individuals experience including emotions (intense feelings that are directed at green HRM), moods (feelings that tend to be less intense, longer-lived, and more diffuse feelings), and attitudes (positive or negative orientations toward green HRM) [45,56]. Based on the affective concepts related to this theory, the current study investigates the other path, i.e., harmonious environmental passion, through which green HRM influences employees’ proactive green behaviors. According to Robertson and Barling [57], harmonious environmental passion refers to a positive emotion that results in an individual wanting to engage in pro-environmental behaviors. Gilal et al. [27] found that green HRM enhances environmental performance via employees’ environmental passion. When employees view the organization that they work for adopting a set of green HRM practices that penetrate various human resource attributions (e.g., rewards for environmentally friendly behaviors, high levels of empowerment given to employees to engage in pro-environmental initiatives and strong employee involvement in green decision-making, take initiative on environmental issues) [58], they are more likely to exhibit a high degree of passion towards these environmentally friendly activities. In this case, green HRM perceived by employees will evoke their harmonious passion for environmental sustainability.

Harmonious passion consists of two key characteristics: the activity that one feels harmoniously passionate about is autonomously integrated into one’s identity and is enjoyable to oneself [59]. Employees who experience a high level of harmonious passion will feel energized and such passion will inspire them to make a difference [60,61] and further motivate employees to engage in activities that are the object of their passion. Research has identified positive effects of harmonious environmental passion on individuals’ attitudes and behaviors. Specifically, Robertson and Barling [57] reveal that employees’ environmental passion is a driver of employees’ environmental behaviors. Afsar et al. [62] reported that employees’ levels of environmental passion serve as a predictor of pro-environmental behavior. Moreover, prior studies [63] suggest that positive emotions such as job satisfaction and happiness can encourage employees to exhibit pro-environmental behaviors in the workplace. In this vein, harmonious environmental passion can also be regarded as a positive emotion that is more likely to encourage employees’ green behaviors. Additionally, research conducted by Dong et al. [64] identifies the mediating role of harmonious passion that connects contextual autonomy support and individual autonomy orientation with employee creativity. In summary, we predict that when employees develop higher levels of harmonious passion for the environment, they feel sense of responsibility and are more likely to be motivated to engage in green behaviors such as voluntary workplace green behavior and green creativity to benefit the organization’s environmental development. Thus, we hypothesize the following:

**Hypothesis** **3a.** *Harmonious environmental passion mediates the relationship between employees’ perceived green HRM and employees’ voluntary workplace green behavior.*

**Hypothesis** **3b.** *Harmonious environmental passion mediates the relationship between employees’ perceived green HRM and employees’ green creativity.*

**Figure 1:**
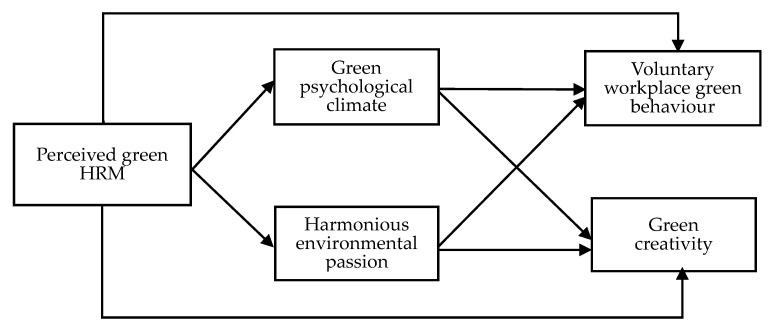
The concept model.

In sum, the hypothesis model is shown in Figure 1.

## 3. Materials and Methods

### 3.1. Participants and Procedure

The sample of firms examined in this study was drawn from the oil and mining industries in China. Compared to other industries, companies of the oil and mining industries generally pose a greater threat to the environment [65]. The oil and mining industry mainly includes oil field and mining services, as well as petroleum and mining equipment manufacturing. It uses oil field and mining as the main business, also providing engineering technical support and solutions for oil and mining exploration and production. Employees in this industry usually work in remote areas next to the oil fields and mines, and they are increasingly adopting highly efficient, energy-saving modern technology. In China, comprehensively improving the level of green and low-carbon recycling, mining technology innovation, and process equipment upgrade has become an urgent task for the high-quality development of the oil and mining industry. Thus, these companies do not simply meet their economic and legal obligations, but consider their green responsibilities to their employees and wider stakeholder groups in society. Before the mass distribution of questionnaires, we contacted four firms located in Hubei province and found that they have implemented green HRM practices to varying degrees. For instance, they have established an energy-saving, emission-reduction and low-carbon office as a daily management organization for energy conservation. Besides, they have established a green training program to ensure that employees fully understand the green standards. Employees are also encouraged to participate in green activities such as process innovation projects, recycling programs, and clean production. Thus, we conducted an onsite investigation and invited frontline employees and their supervisors to participate in our survey. We offered 5 RMB incentive for participation.

A cover letter attached with the questionnaire assured the participants that their participation was voluntary and their responses would be used only for research purposes [66]. To reduce common method variance, data were collected from two sources: employees and their supervisors [67]. We used a code to match employees’ ratings to their supervisors’ responses. Employees completed questionniare included items on their demographic information and perceived levels of green HRM, on their surrounding green psychological climate and on their levels of harmonious environmental passion. A separate questionnaire for supervisors evaluated their subordinates’ behaviors, including their voluntary workplace green behavior and green creativity. The questionnaire was sent onsite three times with a two-week time lag in data collection applied between the measurement of the predictor (perceived green HRM), mediator (green psychological climate and harmonious environmental passion), and outcomes (voluntary workplace green behavior and green creativity).

For the initial survey, we distributed 500 questionnaires, and 100 individuals failed to respond to our survey invitations. After removing incomplete questionnaires, we obtained 358 valid questionnaires yielding a response rate of 71.60%. The majority (57.82%) of the respondents were males. Of the respondents, 19.83% were under 25 years old, 22.90% were 26–35 years old, 22.35% were 36–45 years old, and 34.92% were more than 46 years old. Regarding their work experience, 10.90% had less than 5 years, 21.23% had 5–10 years, 32.12% had 11–15 years, and 35.75% had more than 16 years. Regarding their education level, 16.20% had a secondary technical school degree, 26.26% had a junior college degree, 31.56% had a Bachelor’s degree, and 25.98% had a Master’s degree. As to their occupations, 33.16% engaged in mining work, 21.08% engaged in geological prospecting work, 27.91% engaged in oil and gas field development, and 17.85% engaged in petroleum refining work.

### 3.2. Measures

We followed the standard translation and back-translation procedures [68] to ensure that the survey materials were accurately translated from English into Chinese. We used a 7-point Likert-type scale (from 1 = strongly disagree to 7 = strongly agree) for the participants’ ratings of our survey measures.

#### 3.2.1. Perceived Green HRM

Perceived green HRM was assessed with items taken from Tang et al. [40] (see Appendix A). The 18-item scale represents green HRM in terms of green recruitment and selection, green training, green performance management, green pay and reward, and green involvement. Participants rated their perceptions of their companies’ HRM practices. Items measured included the following: “we attract green job candidates who use green criteria to select organizations”, “we have integrated training to create the emotional involvement of employees in environment management”, “our firm sets green targets, goals and responsibilities for managers and employees”, “in our firm, there are financial or tax incentives (bicycle loams, use of less polluting cars)”, and “in our firm, there is a mutual learning climate among employees for green behavior and awareness in my company”. The coefficient alpha for this scale was measured as 0.83.

#### 3.2.2. Green Psychological Climates

Green psychological climates were assessed with five items taken from Norton et al. [13] (see Appendix B). Each participant rated the extent to which his or her company “is worried about its environmental impact” and “is concerned with becoming more environmentally friendly”. The coefficient alpha for this scale was recorded as 0.83.

#### 3.2.3. Harmonious Environmental Passion

Harmonious environmental passion was assessed with the 10-item scale developed by Robertson and Barling [57] (see Appendix C). Sample items included “I am passionate about the environment” and “I enjoy practicing environmentally friendly behaviors”. The coefficient alpha for this scale was recorded as 0.73.

#### 3.2.4. Voluntary Workplace Green Behavior

Voluntary workplace green behavior was assessed with a measurement developed by Kim et al. [20] (see Appendix D). Supervisors rated the voluntary green workplace behaviors of each member of their work group with 6 items. Behaviors measured included the following: “using stairs instead of elevators when going from floor to floor in the building,” and “recycling reusable things in the workplace”. The coefficient alpha for this scale was recorded as 0.72.

#### 3.2.5. Green Creativity

Green creativity was measured with the 6-item scale developed by Chen and Chang [22] (see Appendix E). Supervisors rated the following behaviors of their employees such as “the members of the green product development project suggest new ways to achieve environmental goals” and “the members of the green product development project promote and champion new green ideas to others”. The coefficient alpha for this scale was recorded as 0.74.

#### 3.2.6. Control Variables

Control variables included each employee’s gender, age, years of work experience and education level because this demographic information may influence individual’s attitude and behavior towards environmental issues based on previous research [11,29].

## 4. Analysis and Results

Table 1 reports the confirmatory factor analysis (CFA), which shows that the proposed five-factor model provides a good fit (χ^2^/df = 1.93, RMSEA (Root Mean Square Error of Approximation) = 0.05, CFI (Comparative Fit Index) = 0.90, IFI (Incremental Fit Index) = 0.90). Table 2 shows the means, standard deviations and correlations among the focal variables. Perceived green HRM is positively related to green psychological climates, harmonious environmental passion, voluntary workplace green behavior and green creativity (r = 0.14, *p* < 0.01; r = 48, *p* < 0.01; r = 33, *p* < 0.01; r = 24, *p* < 0.01, respectively).

We applied the PROCESS method [69] to test the main effect of perceived green HRM on voluntary workplace green behavior and green creativity. The results of a Bootstrap 5000 test are shown in Table 3. The overall effect of perceived green HRM on voluntary workplace green behavior is valued at 0.40 (95% CI: 0.28, 0.52), supporting Hypothesis 1a. Similarly, the overall effect of perceived green HRM on green creativity is valued at 0.30 (95% CI: 0.17, 0.44), supporting Hypothesis 1b. After controlling for the mediating effect of green psychological climates and harmonious environmental passion, the direct effect of perceived green HRM on voluntary workplace green behavior is valued at 0.30 (95% CI: 0.17, 0.44), showing that the direct effect of perceived green HRM on voluntary workplace green behavior is significant. The direct effect of perceived green HRM on green creativity is valued at 0.10 (95% CI: −0.04, 0.24), showing that the direct effect of perceived green HRM on green creativity is non-significant.

We also applied the PROCESS method [69] to test the indirect effect of perceived green HRM on voluntary workplace green behavior and green creativity. The results of a Bootstrap 5000 test are shown in Table 4 and Table 5. As Table 4 shows, the mediating effect of green psychological climate between perceived green HRM and voluntary workplace green behavior is 0.02 (95% CI: 0.001, 0.06), supporting Hypothesis 2a. The mediating effect of harmonious environmental passion between perceived green HRM and voluntary workplace green behavior is 0.08 (95% CI: 0.01, 0.15), supporting Hypothesis 3a. When comparing the two mediating effects, the confidence interval of difference is −0.06 (95% CI: −0.14, 0.03), showing that the difference is non-significant. Similarly, as Table 5 shows, the mediating effect of green psychological climate between perceived green HRM and green creativity is 0.01 (95% CI: −0.004, 0.04), which does not support Hypothesis 2b. The mediating effect of harmonious environmental passion between perceived green HRM and green creativity is 0.19 (95% CI: 0.11, 0.28), supporting Hypothesis 3b. When comparing these two mediating effects, the confidence interval of difference is −0.18 (95% CI: −0.28, −0.10), showing that the difference is significant.

Besides, combining Table 3 with Table 4 and Table 5, we can conclude that green psychological climates and harmonious environmental passion partially mediate the relationship between perceived green HRM and voluntary workplace green behavior while harmonious environmental passion fully mediates the relationship between perceived green HRM and green creativity.

## 5. Conclusions and Implication

The main purpose of this study was to examine the relationship between employees’ perceived green HRM and their proactive outcomes (including voluntary workplace green behavior and green creativity) and to explore underlying mechanisms. In line with our expectations, employees’ perceived green HRM positively impacts their voluntary workplace green behavior and green creativity. This finding is in line with past research showing that green HRM has a direct impact on employee outcomes [11,24,26]. Moreover, green psychological climates and harmonious environmental passion were found to partially mediate the relationship between employees’ perceived green HRM and voluntary workplace green behavior while harmonious environmental passion was found to fully mediate the relationship between perceived green HRM and green creativity. These finding are consistent with previous research showing that it is useful to cultivate employees’ green psychological climates and harmonious environmental passion to improve their outcomes [13,57]. Employees’ green psychological climates only partially mediate the relationship between perceived green HRM and voluntary workplace green behavior, which may be because employees are generally motivated to exhibit behaviors that are consistent with their perceptions (cognition) of organizational practices [13] when their emotions (affection) regarding creativity have not been evoked. When employees experience positive emotions (i.e., harmonious environmental passion), they are more likely to become passionate about something of organizational and social importance (e.g., green creativity) to achieve environmental sustainability [57].

### 5.1. Theoretical and Practical Implications

The findings of this study make important contributions to the literature in three respects. First, past research suggests that HRM practices might not directly influence employee outcomes in the workplace but may indirectly do so through certain paths or psychological processes [19]. Despite the considerable volume of literature centered on the effect of green HRM, there is still much to be learned. Specifically, existing research investigated the influence mechanism of green HRM from the theory of planned behavior (TPB) [24], supplies-values fit theory [26,27], social identity and stakeholder theory [11,28], social exchange theory [29], Ability-Motivation-Opportunity (AMO) and contingency theory [30,31], and most of them focused on cognitive perspective of psychological process. Our research therefore contributes to these streams of research and enriches the literature on the psychological processes (i.e., both cognitive and affective paths) that shape individuals’ green behaviors, including employees’ green psychological climates and harmonious green passion. In responding to Renwick et al.’s [12] call for a better understanding of the underlying mechanisms that operate between organizations’ practices and employees’ green behaviors, our research fills a gap in the existing literature and thus makes an indispensable contribution theoretically. Secondly, our study contributes to the literature on environmental management by investigating how individual green behaviors could be promoted in the workplace. While the current literature has documented the antecedents of employees’ green behaviors, including individuals’ values and behavioral intentions [13], corporate environmental responsibility [14], corporate social responsibility [15], daily affect [16], and transformational leadership [17], few works have focused on the role of formal organizational context such as employees’ perceived green HRM. As HRM practices take care of systems and processes to influence employees in an orderly manner on a bigger scale [70], by identifying green HRM as an important management style that contributes to employees’ green behaviors, this study enhances our understanding of human and organizational elements of environmental management and especially of the antecedents of individuals’ proactive green behaviors in the workplace.

As a key practical implication of this study, we find that employees’ perceived green HRM may help organizations stimulate their proactive environmentally oriented behavior. To achieve an organization’s green goals and elicit broader positive employees’ attitudes and behaviors, organizations should hire employees with strong environmental sensibilities and develop training programs to enhance employee skills for effectively undertaking green activities and enhancing green cognition. Organizations should also provide green performance indicators to performance management systems and appraisals. Further, organizations should offer green rewards to employees and involve employees in problem-solving and decision-making regarding green issues. In addition, our findings show that employees’ perceived green HRM influences their outcomes through difference paths. Voluntary workplace green behavior is stimulated by both green psychological climates and harmonious environmental passion. Green creativity is only stimulated by harmonious environmental passion. As green creativity is connected to the maintenance of core competencies of an organization and is very important for firm growth, managerial attention should focus on ways to foster and develop employees’ positive emotions, including their levels of harmonious environmental passion. For example, organizations may cultivate employees’ harmonious environmental passion by strengthening their sense of psychological ownership (e.g., green empowerment and autonomy) and may provide guidance on issues concerning creativity to enhance their employees’ positive views of their organizations.

### 5.2. Limitations and Directions for Future Research

Several limitations of our study can guide future research. Firstly, the data collection process was all carried out in a single location and the data from the oil and mining industry has limited representativeness. As such, it may be necessary to explore how green HRM is implemented in different parts and different industries in China. The present work might be extended to other geographic and industrial settings, both in China and cross-culturally. Secondly, we only tested two mediating effects of cognitive- and affective-based variables on relations between employees’ perceived green HRM and their proactive green behaviors. In order to respond to the call for the development of the psychology of human behaviors and cognitive processes in complex environments [71], future works should explore other potentially mediating processes and the boundary conditions (i.e., task complexity) through which relationships between employees’ perceived green HRM and their proactive green behaviors can be strengthened or weakened. Thirdly, consider the literature on general HRM which strongly emphasizes that culture affects people’s aspirations, attitudes, and behavior. Environmental (or green) organizational culture, which reflects how important environmental problems are to the organization, serve as invisible guiding principles of an employee in the workplace [72]. Future research is recommended to consider the role of green culture when examine the visible green practices. In addition, this study only focused on greenery and green model. Future research is thus suggested to explore more the biological aspects of greenery to see if there are other methods, such as brief relaxation practice, that are more effective to improve cognitive performance of employees [73]. Lastly, the current study mainly talked about psychological theories and did not study the biological aspects of greenery. However, there is also research evidence examining the greenery issue from the biological perspective, proving that certain horticultural therapy and greenery can be helpful in improving employees’ physical health and mental health, thus activating more proactive green behaviors [74]. Future research is suggested to study more about the biological mechanism through which proactive green behaviors are more likely to be aroused.

## Figures and Tables

**Table 1:** Confirmatory factor analysis results for model comparisons.

Model Types	χ^2^/df	Change χ^2^	RMSEA	CFI	IFI
Five-factor model (expected model)	1.93	-	0.05	0.90	0.90
The best four-factor model (1: Perceived green HRM; 2: GPC; 3: HEP; 4: VGB + GC)	2.16	198.05 ***	0.06	0.87	0.88
The best three-factor model (1: Perceived green HRM; 2: GPC + HEP; 3: VGB + GC)	2.49	479.90 ***	0.07	0.84	0.84
The best two-factor model (1: Perceived green HRM + VGB + GC; 2: GPC + HEP)	2.82	758.59 ***	0.07	0.80	0.81
One-factor model (1: Perceived green HRM + VGB + GC+ GPC + HEP)	3.22	1101.91 ***	0.08	0.76	0.76

Notes: *** *p* < 0.01; GPC = Green psychological climate; HEP = Harmonious environmental passion; VGB = Voluntary workplace green behavior; GC = Green creativity.

**Table 2:** Descriptive statistics and correlations of variables.

Variables	1	2	3	4	5
1. Perceived green HRM	1				
2. Green psychological climate	0.14 **	1			
3. Harmonious environmental passion	0.48 **	0.38 **	1		
4. Voluntary workplace green behavior	0.33 **	0.20 **	0.29 **	1	
5. Green creativity	0.24 **	0.19 **	0.37 **	0.30 **	1
Mean	4.88	4.04	4.68	4.60	4.49
S.D.	0.61	1.07	0.63	0.75	0.77

Notes: ** *p* < 0.01 (Two tailed).

**Table 3:** Total and direct effects.

Model Pathways	Effect	SE	BC95% CI Lower Upper
Total effects			
Perceived green HRM→VGB	0.40	0.06	(0.28, 0.52)
Perceived green HRM→GC	0.30	0.07	(0.17, 0.44)
Direct effects			
Perceived green HRM→VGB	0.30	0.07	(0.17, 0.44)
Perceived green HRM→GC	0.10	0.07	(−0.04, 0.24)

Notes: GPC = Green psychological climate; HEP = Harmonious environmental passion; VGB = Voluntary workplace green behavior; GC = Green creativity; SE = standard errors; CI = confidence intervals.

**Table 4:** Indirect effects of perceived green HRM and voluntary workplace green behavior.

Model Pathways	Effect	SE	BC95% CI Lower Upper
Total indirect effects Mediating effect of GPC	0.10 0.02	0.04 0.02	(0.03, 0.17) (0.001, 0.06)
Mediating effect of HEP	0.08	0.04	(0.01, 0.15)
GPC vs. HEP	−0.06	0.05	(−0.14, 0.03)

Notes: GPC = Green psychological climate; HEP = Harmonious environmental passion; GPC vs. HEP = GPC − HEP, SE = Standard errors; CI = Confidence intervals.

**Table 5:** Indirect effects of perceived green HRM and green creativity.

Model Pathways	Effect	SE	BC95% CI Lower Upper
Total indirect effects Mediating effect of GPC	0.20 0.01	0.04 0.01	(0.12, 0.29) (−0.004, 0.04)
Mediating effect of HEP	0.19	0.04	(0.11, 0.28)
GPC vs. HEP	−0.18	0.05	(−0.28, −0.10)

Notes: GPC = Green psychological climate; HEP = Harmonious environmental passion; GPC vs. HEP = GPC − HEP, SE = Standard errors; CI = Confidence intervals.

## Data Availability

The data presented in this study are available on request from the corresponding author.

## Appendix A. Items Used to Measure Perceived Green HRM

### Appendix A.1. Green Recruitment and Selection

1. We attract green job candidates who use green criteria to select organizations;
2. We use green employer branding to attract green employees;
3. Our firm recruits employees who have green awareness;

### Appendix A.2. Green Training

1. We develop training programs in environment management to increase environmental awareness, skills and expertise of employees;
2. We have integrated training to create the emotional involvement of employees in environment management;
3. We have green knowledge management (link environmental education and knowledge to behaviors to develop preventative solutions);

### Appendix A.3. Green Performance Management

1. We use green performance indicators in our performance management system and appraisals;
2. Our firm sets green targets, goals and responsibilities for managers and employees;
3. In our firm, managers are set objectives on achieving green outcomes included in appraisals;
4. In our firm, there are dis-benefits in the performance management system for non-compliance or not meeting environment management goals;

### Appendix A.4. Green Pay and Reward

1. We make green benefits (transport/travel) available rather than giving out pre-paid cards to purchase green products;
2. In our firms, there are financial or tax incentives (bicycle loans, use of less polluting cars);
3. Our firm has recognition-based rewards in environment management for staff (public recognition, awards, paid vacations, time off, gift certificates);

### Appendix A.5. Green Involvement

1. Our company has a clear developmental vision to guide the employees’ actions in environment management;
2. In our firm, there is a mutual learning climate among employees for green behavior and awareness in my company;
3. In our firm, there are a number of formal or informal communication channels to spread green culture in our company;
4. In our firm, employees are involved in quality improvement and problem-solving on green issues;
5. We offer practices for employees to participate in environment management, such as newsletters, suggestion schemes, problem-solving groups, low-carbon champions and green action teams [40].

## Appendix B. Items Used to Measure Green Psychological Climates

1. (Our company) is worried about its environmental impact;
2. (Our company) is interested in supporting environmental causes;
3. (Our company) believes it is important to protect the environment;
4. (Our company) is concerned with becoming more environmentally friendly;
5. (Our company) would like to be seen as environmentally friendly [13].

## Appendix C. Items Used to Measure Harmonious Environmental Passion

1. I am passionate about the environment;
2. I enjoy practicing environmentally friendly behaviors;
3. I enjoy engaging in environmentally friendly behaviors;
4. I take pride in helping the environment;
5. I enthusiastically discuss environmental issues with others;
6. I get pleasure from taking care of the environment;
7. I passionately encourage others to be more environmentally responsible;
8. I am a volunteered member of an environmental group;
9. I have voluntarily donated time or money to help the environment in some way;
10. I feel strongly about my environmental values [57].

## Appendix D. Items Used to Measure Voluntary Workplace Green Behavior

1. (This member) avoiding unnecessary printing to save papers;
2. (This member) using personal cups instead of disposable cups;
3. (This member) using stairs instead of elevators when going from floor to floor in the building;
4. (This member) reusing papers to take notes in the office;
5. (This member) recycling reusable things in the workplace;
6. (This member) sorting recyclable materials into their appropriate bins when other group members do not recycle them [20].

## Appendix E. Items Used to Measure Green Creativity

1. The members of the green product development project suggest new ways to achieve environmental goals;
2. The members of the green product development project propose new green ideas to improve environmental performance;
3. The members of the green product development project promote and champion new green ideas to others;
4. The members of the green product development project develop adequate plans for the implementation of new green ideas;
5. The members of the green product development project would rethink new green ideas;
6. The members of the green product development project would find creative solutions to environmental problems [22].
